# Morphological Transformation in Polymer Composite Materials Filled with Carbon Nanoparticles: Part 2—Thermal and Mechanical Properties

**DOI:** 10.3390/ma15155094

**Published:** 2022-07-22

**Authors:** Elena Ivan’kova, Gleb Vaganov, Elena Popova, Vladimir Yudin

**Affiliations:** 1 Institute of Biosystems and Biotechnology, Peter the Great St. Petersburg Polytechnic University, Polytechnicheskaya 29, 195251 St. Petersburg, Russia; 2 Institute of Macromolecular Compounds of Russian Academy of Sciences, V.O., Bol’shoy pr. 31, 199004 St. Petersburg, Russia; glebvaganov@mail.ru (G.V.); men682003@mail.ru (E.P.); yudin@hq.macro.ru (V.Y.)

**Keywords:** HDPE, carbon nanodiscs, melt extrusion, fibers, DSC, TGA, mechanical properties, creep

## Abstract

HDPE-based composite fibers filled by original and annealed carbon nanodiscs (oND and aND, respectively) were prepared by melt extrusion technology with high-temperature orientational drawing up to draw ratio DR = 8. The thermal properties of the obtained fibers were investigated by DSC and TGA methods. It was shown that the nanofillers can be influenced by high temperatures, at which the molecular mobility in the interlamellar regions became active, while the melting point and the crystallinity degree of the samples were not affected. Short- and long-term mechanical properties of the nanocomposite fibers were studied as well. Very rare mechanical testing of the knotted fibers was carried out and, as a result, a decrease of the knot strength up to 35% was detected. It was also revealed that the carbon nanodiscs do not reinforce the composite fibers and play a negative role in the creep processes, while the Young’s modulus can be improved by 2 times for the oriented samples.

## 1. Introduction

Thanks to low production cost, chemical resistance and good mechanical properties, high-density polyethylene (HDPE) has been extensively investigated over the last number of decades and widely applied in many fields. Possessing simple chemical structure, HDPE is the most studied polymer both in pure form and as a matrix of the composite materials.

The present work is a continuation of the research of the melt-extruded composite HDPE-based fibers filled with carbon nanoparticles in a shape of nanodiscs, namely, original (oND) and annealed (aND) ones [1]. It was revealed that both types of the nanoparticles can be well dispersed in the HDPE matrix and aligned along the extrusion direction of the composite fibers. However, both types of the carbon nanodiscs demonstrate weak adhesion to the HDPE matrix. Moreover, the annealed nanoparticles (aND) possess a nucleation ability that leads to the growing of the polyethylene transcrystallites, normally to the discs’ surface.

It was also found that, in the pure HDPE fiber and in the composite fiber filled by the amorphous oND, two types of the texture (A or AB) are revealed by WAXS methods, while in the HDPE-based fiber with high amounts of aND, the C-texture becomes dominant. The conclusion was made that the round nanoparticles are capable of transforming the predominant orientation of the morphological units in the composite fibers. This is ascribed to the change in the rheological properties of the composite melt (i.e., an increase in its viscosity) and to the changes of the crystallization conditions of the polymer. Nevertheless, the nanodiscs do not hinder the orientation of the HDPE macromolecules along the fiber axis during high-temperature drawing.

A large number of publications are related to the research of morphological changes that occur in a polymeric matrix when different nanofillers are incorporated into it (in most cases, nanotubes or nanofibers are used). In a large review [2], special attention is paid to the formation of hybrid crystal structures in the polymer composite materials. At the same time, it is emphasized that the mechanism of formation of various types of hybrid crystalline regions (transcrystallites, shish-kebabs, hybrid spherulites, etc.) is not clear yet. The influence of the hybrid crystals on the mechanical properties of the composite materials is also ambiguous. Most authors describe a noticeable increase in adhesion between the nanoadditives and the polymer matrix, which leads to an improvement in the mechanical characteristics of the obtained composite material. Nevertheless, there are data indicating that hybrid crystallization in practice may not have a significant effect on the strength and Young’s modulus of the composites. A number of authors show that the presence of the hybrid crystals can not only improve mechanical properties, but also affect other macroscopic properties (thermal, barrier, etc.) [3,4,5,6], which requires further intensive research.

Considerably less attention is paid to the study of long-term mechanical characteristics (creep)—apparently, this is due to the long duration of such testing [7]. At the same time, low creep resistance can severely limit the use of any structural materials, including the polymers.

Detailed investigation of variation in the thermal and mechanical properties of the HDPE-based composite fibers doped with the original and annealed carbon nanodiscs is a main goal of the work described in the present article.

## 2. Materials and Methods

High-density polyethylene (HDPE; M_w_ = 210,000 g/mol) synthesized in the laboratory has been used as a matrix to produce nanocomposite fibers by melt technology. Two kinds of nanoparticles, i.e., carbon nanodiscs in original state (oND) and ones exposed to an additional annealing at 2500 °C (aND) have been chosen as fillers. Both types of nanodiscs have a wide range of sizes (0.3–4.8 µm). The concentrations of both types of the nanodiscs have been varied, namely, 1, 3, 5 and 10 wt.%.

Dispersing of the filler in the polymer melt as well as the nanocomposite fibers extrusion was carried out by a twin-screw microcompounder DSM Xplore (Xplore Instruments, Sittard, The Netherlands) equipped with a special fiber preparation unit (DSM Film Device Machine, Xplore Instruments, Sittard, The Netherlands). The components were mixed for 5 min at 160 °C at a screw rotation speed of 50 rpm. After that, formation of the nanocomposite fibers occurred via fixed circular die followed by cooling by a flat jet of compressed air using the so-called “air knife” (*p* = 3.9 Pa) just after leaving the spinneret. The cooled fibers were wound on a receiver coil at a constant speed. Finally, the undrawn fibers containing different concentrations of two types of the carbon nanodiscs were obtained.

The prepared fibers were subjected to high-temperature orientational drawing up to draw ratio DR = 8 using special home-made equipment. Drawing was carried out in two stages: the fibers were initially drawn up to DR = 4 at 65 °C, and then another 2 times at 100 °C.

The thermal properties of the HDPE-based fibers were investigated by the differential scanning calorimetry (DSC) method with the aid of a DSC 204 F1 device (NETZSCH, Selb, Germany). The experiments were made in the temperature range 30–170 °C at the heating rate of 10 °C/min, in an inert Ar atmosphere. For calculation of the degree of crystallinity of the prepared samples, the value of melting enthalpy (*ΔH*^0^_m_) as 294 J/g [8] was used, which had been earlier found for HDPE with the crystallinity degree of 100%.

Short-term mechanical tests were carried out using a universal tensile machine INSTRON 5943 (Instron, High Wycombe, UK) using special clips according to the ISO 527 Standard at the tensile rate of 10 mm/min. The fibers’ basic length was equal to 100 mm. Totally, 10 samples for each fiber type were examined. Tensile strength (*σ*_br_, MPa) and the strain at break (*ε*_br_, %) were measured from the obtained stress–strain curves. The measurement error did not exceed 15%.

## 3. Results and Discussion

### 3.1. DSC and TGA

**Figure 1:**
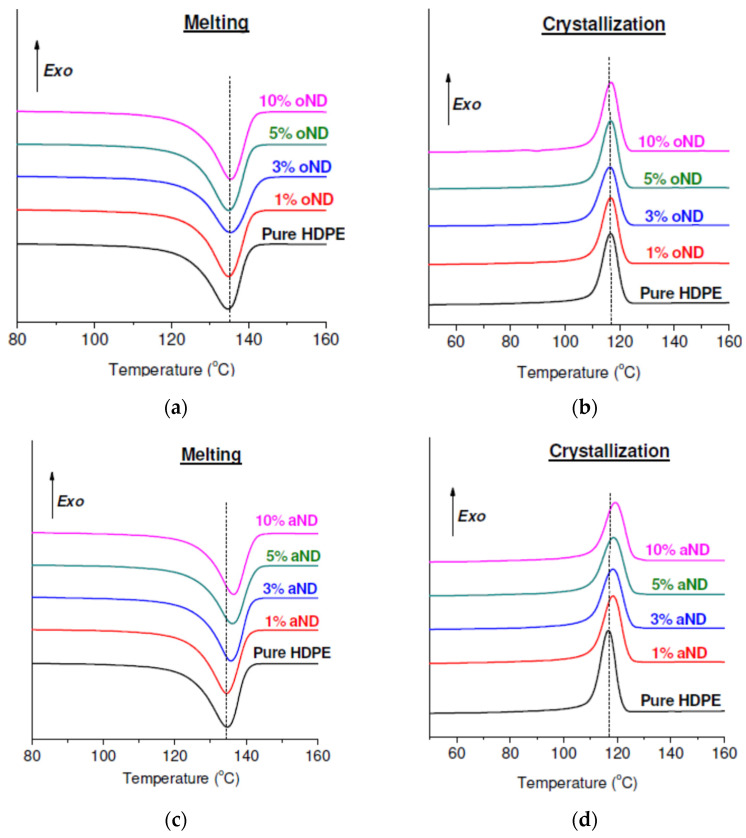
Thermograms of melting (left) and crystallization (right) processes of the unoriented fibers with the addition of oND (**a**,**b**) and aND (**c**,**d**) nanoparticles.

Figure 1 shows, as an example, the melting (a,c) and crystallization (b,d) curves for unoriented fibers with the addition of original and annealed nanodiscs.

**Figure 2:**
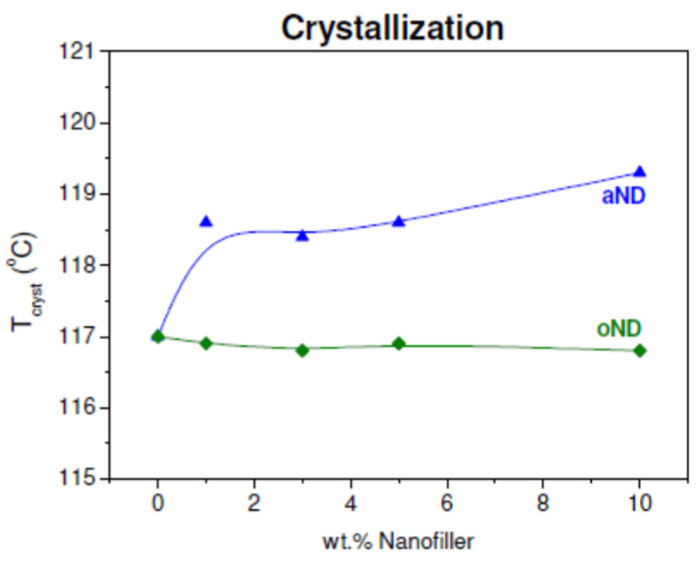
Dependence of the crystallization temperature on the nanofiller content.

At first, it would be reasonable to dwell on the analysis of crystallization curves. It is clearly seen that the maxima of the crystallization peaks for the HDPE-based fibers filled with oND (Figure 1b) do not demonstrate any shift along the temperature scale. However, such a situation was not observed for all composite fibers. The crystallization peaks of the fibers doped with aND, however, are shifted to a higher temperature region compared to pure HDPE fiber (Figure 1d). For clarity, we plotted the dependence of the crystallization temperatures on the filler content for all the samples studied (see Figure 2).

Looking at Figure 2, the difference in the effect between the original and annealed nanodiscs on the crystallization processes occurring during cooling of the HDPE-based sample is immediately obvious: even at low concentrations, the annealed carbon nanodiscs (aND) lead to a sharp increase in *T*_cryst_. This fact indicates that the aND nanoparticles facilitate and accelerate the crystallization process of the HDPE matrix located around them. In other words, this type of the nanodisc exhibits nucleating ability, which further leads to the formation of transcrystalline polymer regions around them that were already revealed by scanning electron microscopy (SEM) and discussed earlier [1]. At the same time, it should be noted that the original nanodiscs (oND) do not possess such ability with respect to HDPE. All this is additional confirmation for the WAXS data reported in [1] that the aND fillers are capable of a change in the predominant orientation (texture) of the crystalline regions of the polymer.

Let us now return to the DSC data on the melting of the fibers studied. By analyzing the melting curves, it is possible to determine and calculate a number of parameters, the evaluation and comparison of which will provide important information about the properties of the nanocomposite samples and the processes occurring in them during heating: melting temperature (*T*_m_), the temperatures of the beginning and end of the melting process (*T*_1_ and *T*_2_), melting enthalpy (Δ*H*_m_) together with the degree of crystallinity calculated from it (*χ*, %), the temperature at which the interlamellar molecular mobility occurs (*T*_am_), etc.

**Figure 3:**
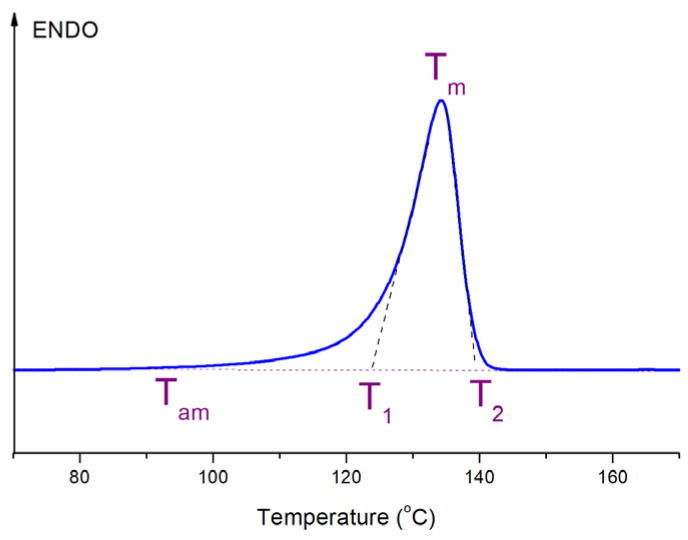
An example of the melting thermogram of the pure HDPE undrawn fiber.

All melting curves obtained for all types of the pure HDPE and the composite fibers before and after orientation drawing were carefully processed and analyzed. One of them is presented, as an example, in Figure 3. To save space, we will not present the dependences of all parameters of the melting peaks on the content and type of the nanofiller, but will dwell only on the most significant results, from our point of view.

**Figure 4:**
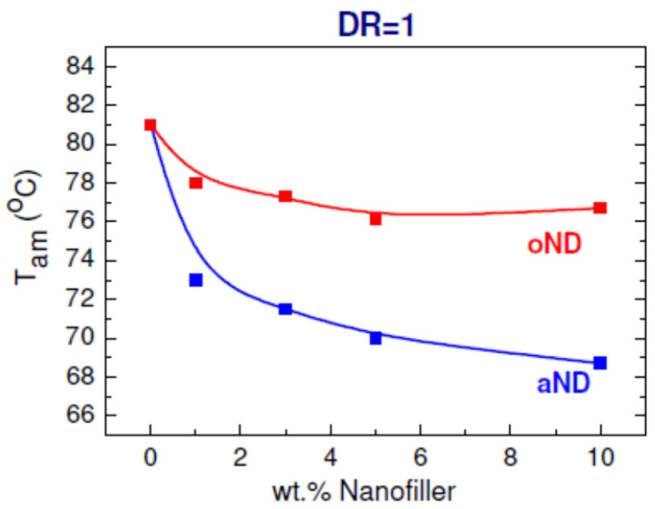
Dependence of the temperature *T*_am_ on the filler content in the unoriented composite fibers based on HDPE.

Analyzing the data obtained by DSC, the dependences of temperature *T*_am_ on the content of the nanofillers is plotted (see Figure 4) for unoriented samples (DR = 1). The temperature *T*_am_ is determined according to the literature data [9] as the temperature at which molecular mobility occurs in the interlamellar places (i.e., when the melting of the crystallites themselves has not yet begun). The fact that *T*_am_ decreases with an increase in the proportion of the nanoparticles in the unoriented fibers indicates an enhancing amorphization of the filled samples, that could then negatively affect the strength of the HDPE-based fibers with DR = 1. Nevertheless, it can be assumed that such an earlier unfreezing of the macromolecular mobility in the amorphous regions facilitates the further rearrangement of the structure from the lamellar to the fibrillar one during the process of orientational drawing, which should ultimately lead to an improvement in the tensile mechanical properties of the oriented fibers (DR = 8). It should also be noted that the fiber-drawing temperature was 65 °C, which is only slightly lower than *T*_am_ for the unoriented highly filled (≥5 wt.%) fibers with the aND nanoparticles. At the same time, for the unoriented fibers containing a small amount of filler (and especially for the pure HDPE), the difference between *T*_am_ and *T*_draw_ reaches 12 °C. Thus, it can be concluded that, despite the HDPE lamellae possessing the C-texture and being the most inconvenient for the recrystallization and unfolding of the macromolecular folds, it is easier for such lamellae to unfold along the directions of the orientation and recrystallize to form fibrils due to increased molecular mobility in the interlamellar regions. In addition, it is known [8,10] that the melting of polymer crystallites starts from the side surfaces and from the defect sites (chain end, folds, etc.)—it occurs by separating the chain sections in the direction perpendicular to the *c*-axis of the crystallite. At the same time, the greater the number of the introduced aND nanoparticles is, the more transcrystallites adjoin them with their side faces, while the cohesive interaction between the elements of the polymer chains decreases. Consequently, in view of the low adhesion between the nanofiller and the HDPE matrix [1], it will be easier for such transcrystallites to turn along the orientation axis during the orientational drawing process following the unfolding of their chain folds. All this will undoubtedly affect the quality of the resulting fibrillar structure and further contribute to the increase in the strength of these fibers after orientation drawing (compared to the pure HDPE). However, it should not be forgotten that the filler nanoparticles having poor adhesion to the polyethylene matrix can play the role of defects (i.e., they can practically be considered as pores). Therefore, their large number can adversely affect the behavior of the composite material in the field of mechanical forces, leading to the fact that the processes of destruction will begin to prevail over the processes of hardening—this, in our opinion, explains the impossibility of drawing the extremely filled (10 wt.%) composite fibers even up to DR = 8.

In the composite fibers containing the oND nanoparticles, the texture of the crystalline regions in the matrix did not change compared to the pure polymer (the A-texture predominates, which is the most convenient for further unfolding of the macromolecular chain folds and transformation of the lamellae into the fibrils) [1]. However, for the concentration of these nanofillers more than 5 wt.%, the temperature *T*_am_ decreases only by ~7 °C (compared to the pure HDPE). As was shown earlier, such HDPE lamellae are mostly oriented along the fiber axis and also parallel to the surfaces of the nanodiscs. In addition, in these samples, the increase in the content of the nanoparticles will lead to the increase in the number of the lamellae surrounding these particles, and, thus, is less associated with the rest of the matrix. Moreover, such lamellae directly adjacent to the surfaces of the nanodiscs can easily slide along the atomically smooth carbon surfaces of the nanoparticles. Ultimately, all this will also facilitate the rotation of some inconvenient lamellar formations along the fiber axis during orientation drawing and will further improve the quality of the fine structure of the fibrils in the oriented polymer and, as a consequence, should increase the composite fiber strength.

We believe that the results described above can explain the reason why the melt-extruded composite fibers with oND and aND nanoparticles, having the very different A- and C-textures, demonstrate nearly the same crystalline orientations after drawing up to DR = 8 (their misorientation angle along the fiber axis is very low and is equal to 4.5–5°) [1].

The other very important parameter derived from the DSC melting curves is the melting temperature (*T*_m_). As follows from the processing of the obtained DSC data, the melting temperature in the unoriented (DR = 1) and oriented fibers (DR = 8) changes little (within tenths of the Centigrade) when the nanoparticles of any type and in any amount are introduced (see Table 1). According to the Thomson–Gibbs equation [2], *T*_m_ is related to the size (along the *c*-axis) of the crystalline regions in the polymer as well as to their perfectness degree. Thus, the DSC data confirm the results of the X-ray diffraction analysis that the introduction of the filler nanoparticles has an insignificant effect on the size of the HDPE crystallites [1]. However, for the samples subjected to orientation drawing, *T*_m_ is 5 °C higher than before. This can indicate that the average crystallite size along the *c*-axis in the oriented fibers becomes somewhat larger. Furthermore, it can mean that the fibers’ drawing was effective and the newly formed fibrils consist of rather perfect crystallites.

We also evaluated the degree of crystallinity (*χ,* %) of all the composite fibers before and after orientation drawing (see Table 2). In the unoriented fibers (DR = 1), the introduction of both types of the nanodiscs does not significantly influence the degree of crystallinity. In the oriented samples, *χ*^DR = 8^ also does not depend so much on the content of the original nanodiscs, while it slightly rises in the case that the annealed nanodiscs are used.

It should be also noted that *χ*^DR = 8^ exceeds by 10–30% the degree of crystallinity of the unoriented samples (*χ*^DR = 1^). This means that the restructuring of the lamellar structure into the fibrillar one in the process of orientation drawing was successful.

**Figure 5:**
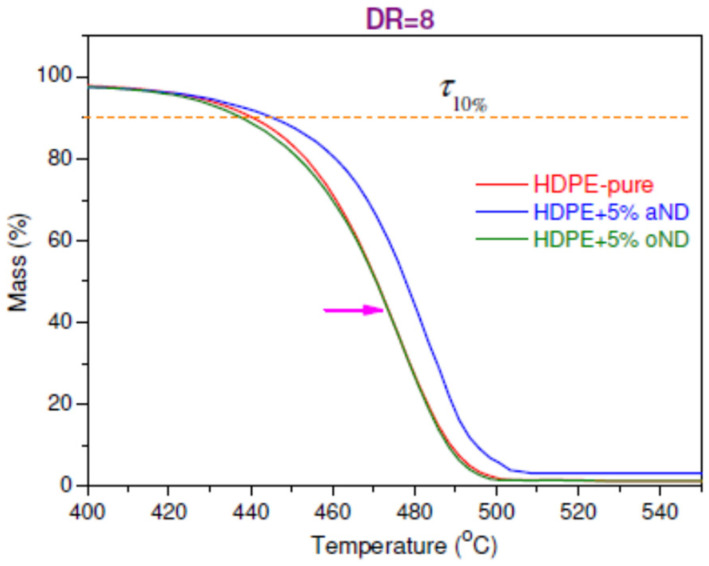
TGA curves of the oriented composite fibers based on HDPE.

Now we can briefly consider the results of thermogravimetric analysis (TGA), which gives us information about the thermal stability of the obtained samples. It is believed that the TGA experiments on the oriented fibers are the most interesting, since such oriented fibers are the most promising in terms of practical use (compared to the unoriented samples). Figure 5 shows melting thermograms of the fibers melt-spun from pure HDPE and the filled (5 wt.%) composite samples (the fibers with intermediate filler contents were also studied).

Based on the presented data, it can be concluded that all the studied fibers are stable up to the temperatures of ~430 °C, after which the destruction processes begin to become more active. From the available TGA data, it is possible to determine the temperatures of the beginning of the samples’ weight loss (*τ*_10%_ is the temperature at which the loss of 10% of the polymer weight occurs) and the temperatures of the maximum degradation rate (*T*_max_—indicated by an arrow in Figure 5). The obtained values are shown in Table 3.

It should be noted that some minimal effect of increasing the heat resistance of composite fibers based on HDPE is provided by the annealed nanodiscs (aND), while the original nanodiscs (oND) do not demonstrate any thermal stability improvement of the HDPE-based composite fibers. It is known that the formation of a rigid polymer network leads to an increase in the thermal stability of compositions. This is in good agreement with our last data on the rheology of the HDPE melts with the same nanofillers [1]. It was clearly shown that the introduction of the annealed carbon nanodiscs into the HDPE matrix leads to a sharp increase in the viscosity at the low shear rate.

### 3.2. Mechanical Properties of Polymer Composite Samples

**Figure 6:**
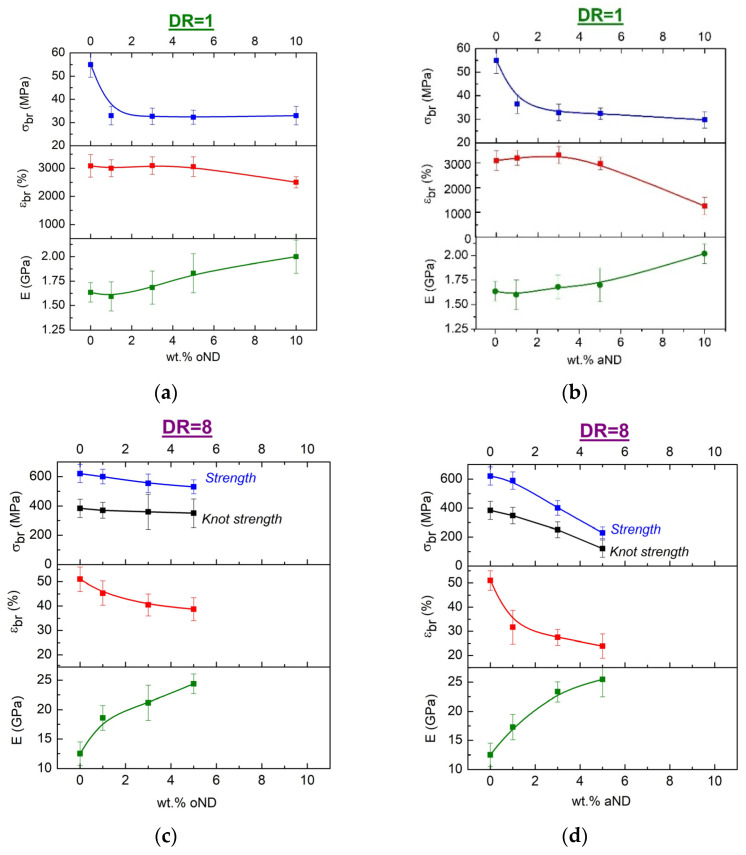
The mechanical characteristics of the composite fibers and dependences on the type and content of the nanofiller: (**a**,**b**) unoriented and (**c**,**d**) oriented samples.

Figure 6 presents the data obtained as a result of the mechanical tests of all the studied samples, i.e., the nanocomposite HDPE-based fibers before and after orientational drawing. The spread of the values of certain measured characteristics did not exceed 15–20% for each test sample, so we did not reflect this on the graphs. It immediately catches the eye that the strength of the fibers in the unoriented state (DR = 1) decreases by about 45% with the introduction of any type of nanodisc.

It should also be noted that the introduction of the filler nanoparticles into the unoriented fibers leads to a noticeable decrease in deformation at break (*ɛ*_br_). The decrease in *ε*_br_ can be almost 3 times in magnitude (from *ε*_br_ = 3050% in pure HDPE to *ε*_br_ = 1000% in the fiber with 10 wt.% of the aND nanofiller). The reason for such a sharp decrease in *ε*_br_ for the carbon nanodiscs may be the fact that the adhesion of these particles to the HDPE matrix is very low, and, as a result, these particles can be considered as numerous defects (pores) inside the composite material, which leads to a decrease in strength and, most importantly, to embrittlement. Even a rounded shape of the nanoparticles, which could slow down the passage of the main crack and prevent the material from becoming sharply brittle, does not help for the investigated composite fibers.

Nevertheless, in regard to such a filler as oND, the deformation before the fiber rupture is reduced by only 6–10%. It is believed that this is due to the fact that the composite HDPE-based fibers filled by the oND have totally different preferential orientation of the lamellar units, compared to the fibers with the aND [1]. The oND nanoparticles do not change the A-texture of the HDPE matrix; hence, the lamellae are oriented along the fiber extrusion direction (i.e., along the fiber axis). As revealed in the previous paper (Part 1), the A-texture of the crystalline parts of the polyethylene matrix is the most comfortable for the macromolecular chains unfolding. It means that the cold drawing can occur in these composite fibers during mechanical tensile testing even at room temperature. As a result, the deformation at break of these samples is nearly the same as for the pure HDPE ones. However, the presence of the high number of nanoparticles (≥5 wt.%) playing the role of numerous defects leads to some embrittlement of the composite fibers.

It is also seen that there is an increase in the Young’s modulus upon the introduction of the nanofillers, and, apparently, this is due to the reinforcing effect of the hard filler nanoparticles.

In the process of orientation drawing up to DR = 8, as expected, the lamellar structure is rearranged into the fibrillar one, which results in a significant strengthening of the fibers compared to the unoriented samples. However, doping of the HDPE-based fibers by increasing the concentration of carbon nanodiscs does not demonstrate any positive effect: both types of the nanodiscs make the composite fibers less strong, especially the introduction of the aND, which leads to a significant drop in the tensile strength. It is known that the strength of any materials is determined by the presence of “weak” places in them—in this case, the arrangement of their disordered areas. The higher degree of connectivity of the structural units in such areas by the tie polymer chains, the more effectively this material will be able to resist destruction (i.e., a higher number of load-bearing sections of the macromolecules are present in it). Looking at the Figure 6c,d, it can be concluded that transformation of the polyethylene lamellae with C-texture into fibrils inside of the composite fibers filled with aND gives, as a result, a pure arrangement of the disordered regions between the crystallites and a low number of taut-tie molecules. Similar effects were also described in the literature [11].

It is worthy to note that the crystallinity degree of the composite fibers did not change very much when the content of the oND and aND nanoparticles increased both in the undrawn and oriented fibers (Table 2). However, the almost constant level of the crystallinity degree cannot be a guarantee of the constant level of the strength of the material doped by the nanoparticles, since the quality of the amorphous regions’ organization will play more important role. A similar effect has been observed earlier on the other polymer composites [12].

As well as for unoriented samples, the deformation at break (*ε*_br_) of all the oriented composite fibers with DR = 8 decreases significantly with an increase in the concentration of the introduced nanoparticles (by a factor of 2). As noted earlier, with a large number of the nanoparticles, for those that are practically not connected with the main matrix, the possibility of material embrittlement increases markedly. The Young’s modulus of all the oriented fibers increases with an increase in the concentration of the fillers that, apparently, can also be explained by the positive reinforcing effect of the rigid nanoparticles.

**Figure 7:**
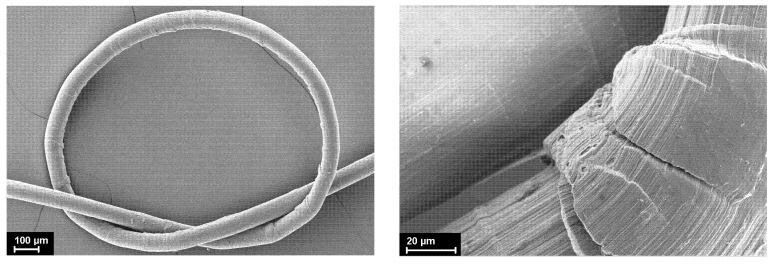
Scanning electron micrographs of the knot of the oriented HDPE-based composite fiber.

In the present work, we decided to conduct a study on the mechanical properties of the oriented composite fibers in a knot. Such studies are rare; despite their simplicity, the information obtained in their course is extremely important. These measurements have not yet been carried out on the composite fibers at all. Thus, we can state the fact that during the loading of the knotted oriented composite fibers, an obvious drop in strength by 30–35% occurs for both types of filler. Moreover, the higher the content of the nanoparticles in the fibers is, the lower the strength of the material decreases. It is known that the HDPE samples are very sensitive to any bends; as a result, numerous kink-bands are formed at the bend and the main crack can propagate along these kink-bands (Figure 7). In the case of the filled fibers based on HDPE, an additional negative contribution is made by the stiff filler particles themselves, which, when the fibers are bent, can destroy the softer HDPE matrix from the inside, leading to a noticeable decrease in the strength.

### 3.3. Study of Long-Term Mechanical Properties (Creep) of Oriented Composite Fibers Based on HDPE

**Figure 8:**
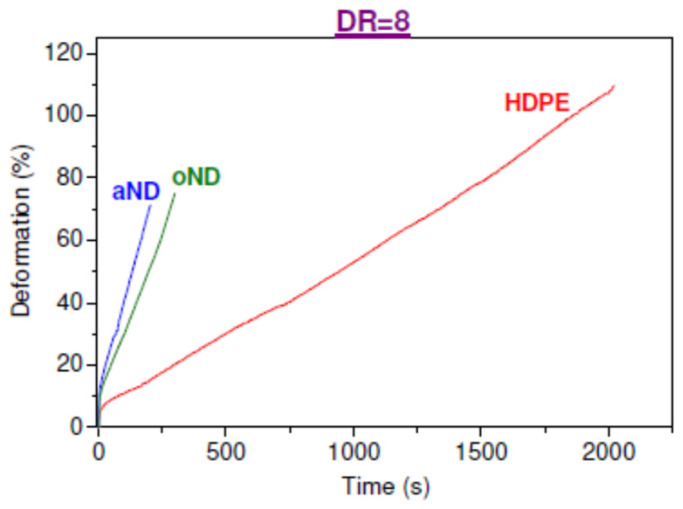
Examples of the creep curves for the HDPE-based oriented composite fibers filled with 5 wt.% nanodiscs.

Now we would like to present the most interesting, from a practical point of view, results on long-term mechanical properties (i.e., creep) of the oriented composite fibers based on HDPE. It is not a secret that the plastic deformation can develop under even small loads, which significantly limits the scope of materials [13,14]. This is especially important for polyethylene, because despite excellent short-term mechanical properties (tensile strength *σ*_br_, strain at break *ε*_br_, etc.), its creep resistance is extremely low, which is one of its significant drawbacks. The creep tests of the HDPE fibers were carried out on a strain relaxometer equipped with a computer system for measuring, controlling and processing the results. The applied static load was 50% of the tensile strength *σ*_br_ for each type of the studied composite fibers—in this case, the samples are in equal conditions with respect to their further destruction. Each sample was loaded until it broke. Curves *ε(t)* were recorded, where *ε* is the deformation accumulated during loading (%) and *t* is the experiment time (sec). A minimum of five experiments were carried out for each fiber type. Figure 8 shows the creep curves for the oriented (DR = 8) composite fibers with the highest possible concentration of nanofiller (5 wt.%).

**Figure 9:**
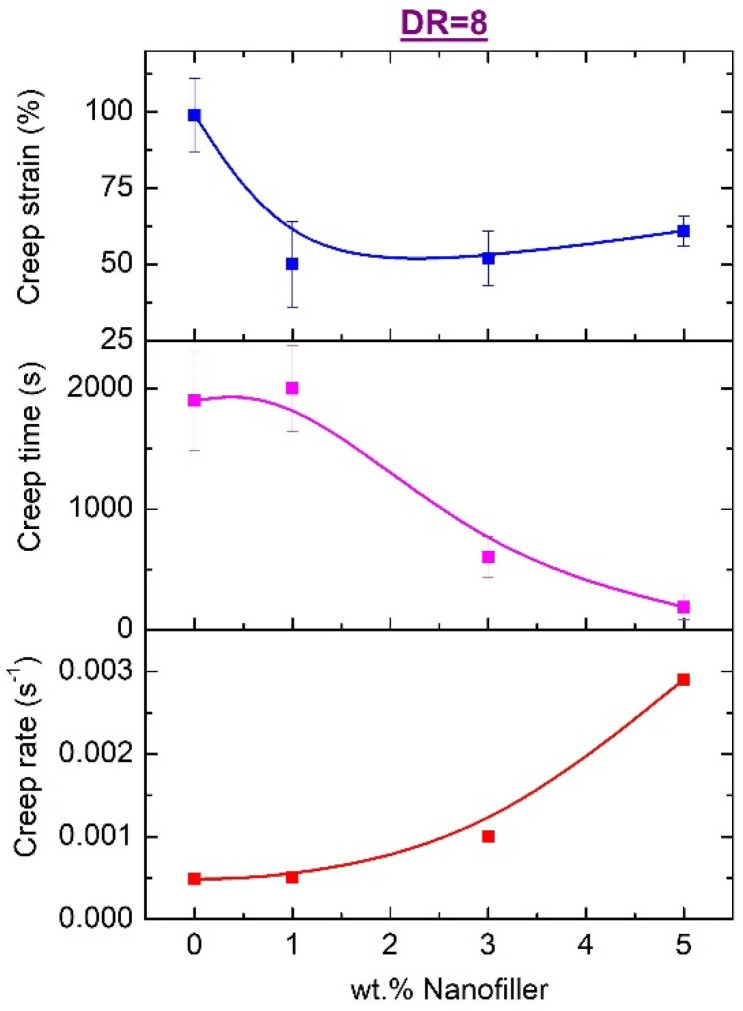
Dependencies of the cumulative creep parameters on the content of the carbon nanodiscs in the oriented composite HDPE-based fibers.

It is obviously seen that the introduction of the filler nanoparticles has an evident effect on the ability of the HDPE fibers to withstand the applied load: Both types of filler, oND and aND, sharply reduce the lifetime of the sample under load as well as the level of deformation accumulated before failure. One of the important indicators is the creep rate (*s*^−1^)—it is determined from the data of the creep curves as the tangent of the slope of the curves at each point. For clarity, we would like to present in Figure 9 the values of the accumulated creep strain before failure, the creep lifetime of the samples under load and the creep rate for these oriented composite fibers.

It is clearly seen that, according to the totality of the data obtained, the most unsatisfactory creep performance was found in the oriented composite fibers with the introduction of both types of the nanodiscs—with a fairly significant decrease in the accumulated strain, the lifetime of such samples drops by almost 10 times and the strain rate increases by almost 6 times (compared to a pure HDPE fiber). Thus, it is clearly shown that the carbon nanodiscs introduced into the HDPE-based fibers have an extremely negative effect on the creep resistance.

It is known that the creep of the fibrillar structure of the oriented polymeric materials depends not so much on their strength as on the possibility of the fibrils slipping relative to each other [15,16,17]. In the case of the composite materials, it is necessary to take into account the relationship between the matrix and the nanofiller, as well as location of the nanoparticles in the composite. The division of the oriented polymer into specific morphological formations, the structure of their interfaces and the degree of bonding of the polymer matrix with the filler nanoparticles should undoubtedly affect the behavior of the oriented polymers and composites in the field of the mechanical forces. Taking into account the earlier-established fact that the adhesion between the filler nanoparticles (oND and aND) and the HDPE matrix is extremely low, it becomes clear why the nanodiscs negatively affect the long-term properties of the studied fibers—these particles are integrated into the interfibrillar space, can have rather large diameters and are practically not connected with the HDPE matrix. This means that due to their presence, the nanodiscs significantly reduce the degree of binding of the HDPE fibrillar elements to each other and facilitate fibrillar slippage. In addition, such nanoparticles act as numerous defects, leading to a noticeable drop in the lifetime of the samples under load. It should also be taken into account that the nanodiscs have transverse dimensions (thickness) of only a few nanometers and an almost atomically smooth surface, which, apparently, leads to a sharp increase in the fiber creep rate.

## 4. Conclusions

In the presented work, the thermal and mechanical properties of HDPE-based fibers doped with the original and annealed carbon nanodiscs (oND and aND) obtained by melt spinning were investigated. The annealed nanoparticles (aND) are found to act as nucleation agents inside the HDPE matrix.

It was observed that the temperature *T*_am_, at which a molecular mobility occurs in the interlamellar places, evidently depends on the type and the content of the introduced nanoparticles. At the same time, the melting point (*T*_m_) and the degree of crystallinity of the studied composite fibers was not affected by the nanoparticle filling.

A small effect of increasing the heat resistance was revealed in the composite HDPE-based fibers with the incorporated annealed nanodiscs (aND), while the original nanodiscs (oND) did not demonstrate any thermal stability improvement of the nanocomposite samples.

It is shown that both types of the carbon nanodiscs are able to increase the Young’s modulus of the undrawn and oriented composite fibers and to make the samples less strong and more brittle. The last fact is explained by the low adhesion between the nanoparticles and HDPE matrix, which results in the dominant role of the destruction processes.

Creep experiments under dead-loading demonstrated sharp drops in the accumulated creep strain and the lifetime of the fibers under applied load, while the creep rate was increased by 6 times.

## Figures and Tables

**Table 1:** Dependence of the melting temperature (*T*_m_) on the nanofiller content in the composite Figure 1. and after (DR = 8) orientation drawing.

Sample	*T*_m_, °C	
	DR = 1	DR = 8
HDPE-pure	135	140
HDPE + 1 wt.% oND	135.1	140.1
HDPE + 3 wt.% oND	135.2	140.1
HDPE + 5 wt.% oND	135.1	140
HDPE + 10 wt.% oND	135.2	-
HDPE + 1 wt.% aND	135.5	140.3
HDPE + 3 wt.% aND	135.6	140.3
HDPE + 5 wt.% aND	135.6	140.2
HDPE + 10 wt.% aND	135.6	-

**Table 2:** Dependence of the degree of crystallinity (*χ*) on the filler content in the composite fibers based on HDPE before (DR = 1) and after (DR = 8) orientation drawing.

Sample	Degree of Crystallinity, %	
	DR = 1	DR = 8
HDPE-pure	60	74.9
HDPE + 1 wt.% oND	60.5	76
HDPE + 3 wt.% oND	60.8	76.1
HDPE + 5 wt.% oND	60.6	76.4
HDPE + 10 wt.% oND	60.1	-
HDPE + 1 wt.% aND	60	77.3
HDPE + 3 wt.% aND	59.1	78.3
HDPE + 5 wt.% aND	58.9	78.3
HDPE + 10 wt.% aND	58.9	-

**Table 3:** The values of the temperature of the beginning of the weight loss of the samples (*τ*_10%_) and the temperature of the maximum degradation rate (*T*_max_) for the oriented composite fibers based on HDPE.

Sample	*τ*_10%_, °C	*T*_max_, °C
HDPE-pure	440.1	475.8
HDPE + 5 wt.% oND	438.4	476.5
HDPE + 5 wt.% aND	445.3	481

## Data Availability

Not applicable.
